# Distinct Roles of Common Genetic Variants and Their Contributions to Diabetes: MODY and Uncontrolled T2DM

**DOI:** 10.3390/biom15030414

**Published:** 2025-03-14

**Authors:** Shadi Bazzazzadehgan, Zia Shariat-Madar, Fakhri Mahdi

**Affiliations:** 1 Department of Pharmacy Administration, School of Pharmacy, University of Mississippi, University, MS 38677, USA; sbazzazz@go.olemiss.edu; 2 Division of Pharmacology, School of Pharmacy, University of Mississippi, Oxford, MS 38677, USA; madar@olemiss.edu

**Keywords:** diabetes mellitus, etiology, genetics, treatment, inflammation, new targets

## Abstract

Type 2 diabetes mellitus (T2DM) encompasses a range of clinical manifestations, with uncontrolled diabetes leading to progressive or irreversible damage to various organs. Numerous genes associated with monogenic diabetes, exhibiting classical patterns of inheritance (autosomal dominant or recessive), have been identified. Additionally, genes involved in complex diabetes, which interact with environmental factors to trigger the disease, have also been discovered. These genetic findings have raised hopes that genetic testing could enhance diagnostics, disease surveillance, treatment selection, and family counseling. However, the accurate interpretation of genetic data remains a significant challenge, as variants may not always be definitively classified as either benign or pathogenic. Research to date, however, indicates that periodic reevaluation of genetic variants in diabetes has led to more consistent findings, with biases being steadily eliminated. This has improved the interpretation of variants across diverse ethnicities. Clinical studies suggest that genetic risk information may motivate patients to adopt behaviors that promote the prevention or management of T2DM. Given that the clinical features of certain monogenic diabetes types overlap with T2DM, and considering the significant role of genetic variants in diabetes, healthcare providers caring for prediabetic patients should consider genetic testing as part of the diagnostic process. This review summarizes current knowledge of the most common genetic variants associated with T2DM, explores novel therapeutic targets, and discusses recent advancements in the pharmaceutical management of uncontrolled T2DM.

## 1. Introduction

**Figure 1:**
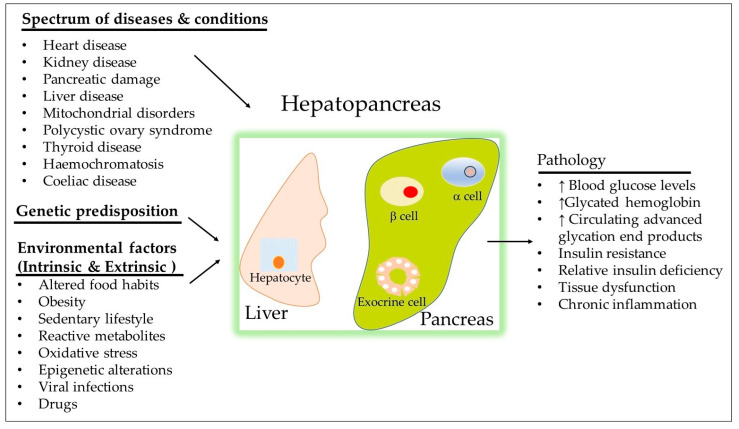
A spectrum of diseases can induce blood glucose alteration and lead to the development of diabetes, which plays a role in pathology. Moreover, the genetic predisposition and environmental factors can significantly impact the normal function of the liver and pancreas, with concomitant clinical outcomes. An up arrow indicates increase and enhancement.

Metabolic mechanisms are intricate networks of biochemical reactions that ensure proper nutrition consumption, energy production, and balance, maintaining cellular, tissue, and organismal integrity and function [1,2]. The hepatopancreatic unit plays a critical role in modulating metabolism and energy flow across tissues, activating complex regulatory genes, cells, and mediators to achieve balance (Figure 1). Consequently, metabolic networks are highly interconnected, sustaining homeostasis [3].

An individual’s metabolism is shaped by genetic factors that influence the stability of metabolic processes over evolutionary time. Accumulating evidence highlights that factors such as age, nutrition, physical activity, and environmental influences significantly affect metabolic function [4,5,6]. Thus, metabolism is influenced not only by genetic factors but also by various extrinsic variables [7,8] (Figure 1).

Excessive nutrition and insufficient energy expenditure are key drivers of T2DM development [9]. Nutritional excess not only elevates insulin secretion but also diminishes insulin’s metabolic effects in certain tissues like the liver, skeletal muscle, and adipose tissue. As depicted in Figure 1, the pathophysiology of T2DM is multifactorial, involving both pancreatic and extrapancreatic factors. Glucose, the body’s primary energy source, is tightly regulated. While normal fasting blood glucose levels typically range between 70 and 80 mg/dL, considerable variability exists even among healthy individuals, largely driven by genetic factors. In general, when glucose disturbances are minor, the homeostatic system restores normal levels to meet bodily needs [10]. However, when the disturbance in glucose homeostasis is significant and sustained, the homeostatic response may develop into a pathological process, resulting in abnormal glucose accumulation. Over time, this can lead to endothelial cell insulin resistance and reduced insulin secretion, and ultimately T2DM.

Cardiovascular (CV) disease, kidney damage, mitochondrial disorders, along with the associated oxidative stress are significant contributors to the high morbidity rates in individuals with T2DM. Conversely, CV disease remains the most severe complication of T2DM. Although significant advances are being made to manage CV disease in recent years, many individuals with diabetes continue to suffer from uncontrolled hyperglycemia, hypertension, and dyslipidemia [11].

**Figure 2:**
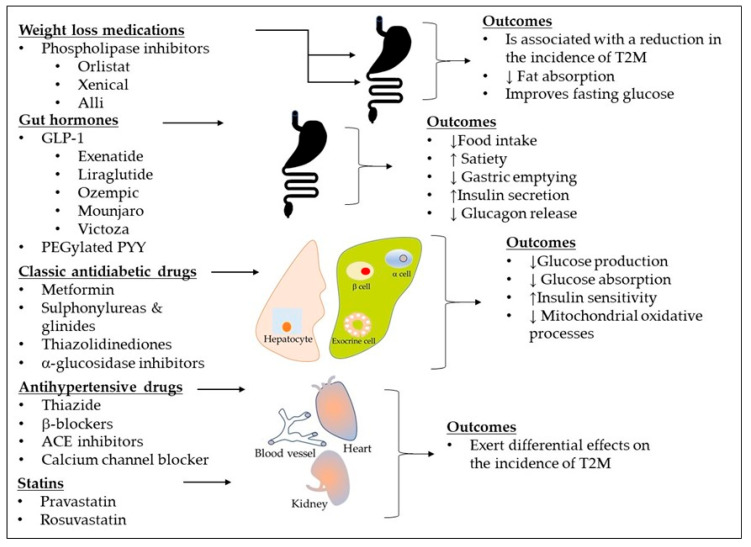
Current strategies to target uncontrolled diabetic patients primarily focus on lifestyle modification combined with medications to achieve target glucose, blood pressure, and cholesterol. An up arrow indicates increase or enhancement, a down arrow shows decrease.

Various pharmacological treatments effectively lower hyperglycemia in T2DM patients and reduce the risk of CV disease and kidney damage (Figure 2). These medications exert their effects through an array of diverse mechanisms, including enhancing insulin secretion from pancreatic beta-cells, promoting weight loss, reducing peripheral insulin resistance, decreasing mitochondrial oxidative stress, and minimizing fat absorption. Despite these treatments, blood glucose remains uncontrolled in many T2DM patients, leading to CVD, renal failure, and blindness [12]. The 2023 American Association of Clinical Endocrinology (AACE) diabetes updates emphasize lifestyle modifications (Figure 1) and highlight the importance of appropriate management of atherosclerotic risk factors like dyslipidemia and hypertension [13]. A complications-centered approach has emerged as a promising alternative to the glucose-centered approach in preventing the progression of prediabetes to diabetes and minimizing organ damage from diabetes and comorbidities like hypertension, obesity, and modifiable risk factors (both intrinsic and extrinsic, as shown in Figure 1. However, the frequency of diabetes-related complications is strangely higher in racial and ethnic minority populations [14].

In addition to age and being overweight, which are well-established independent risk factors for uncontrolled diabetes, several other lifestyle and biological factors contribute to uncontrolled T2DM. These include a strong family history of diabetes [15], ethnicity, gestational diabetes history, elevated blood pressure [16], abnormal blood lipid levels, increased atherogenic cardiovascular risk, and liver disease [17].

Genetic factors may play a more prominent role in predisposing individuals to T2DM [18]. Numerous genetic variants influencing both T2DM [19] and blood glucose levels [18] have been identified. Moreover, some studies highlight the importance of specific genes in determining the inter-individual variability in response to antihyperglycemic treatments [20,21,22].

In October 2024, we conducted an electronic search using PubMed, Medline, U.S. Food and Drug Administration (FDA, fda.gov https://www.fda.gov/drugs/novel-drug-approvals-fda/novel-drug-approvals-2024. Accessed date 15 December 2024. Content current as of: 10 January 2025), ClinicalTrials.gov, and Scopus databases. The search focused on keywords and MeSH terms such as genetic polymorphisms in DNA coding regions and the prevalence of specific polymorphisms across different ethnic groups. This review summarizes and compares whether single nucleotide polymorphisms (SNPs) previously associated with blood glucose levels, diabetes severity, or antihyperglycemic treatment selection (from literature prior to 2020) continue to be relevant.

This review aims to update current knowledge on the genetic and epigenetic variants associated with diabetes risk and prevention, particularly in individuals with uncontrolled T2DM. Additionally, it provides insights into newly approved therapies and those under investigation (Phase 2 and Phase 3) for T2DM treatment. Despite a significant proportion of T2DM patients being treated for their condition, only a small fraction achieve glycemic control, making uncontrolled T2DM a major clinical issue. Therefore, novel treatment strategies are urgently needed to prevent serious complications and reduce the need for hospitalization.

## 2. Definition of Uncontrolled T2DM

Plasma glycosylated hemoglobin A1C (HbA1C) is a key predictor for the progression of prediabetes to T2DM [23], and serves as an important marker for diabetes-related complications [24]. While HbA1C levels are often met with considerable skepticism, they play a central role in the diagnosis and evaluation of diabetes treatment and prognosis [25].

To prevent complications associated with diabetes, the American Diabetes Association (ADA) has established clinical practice guidelines [26]. One of these recommendations is the active monitoring of HbA1C and glucose parameters for optimal assessment of glycemic status. The 2023 AACE diabetes updates emphasizes a complication-centric approach, going beyond the focus on glucose levels alone [13]. According to the ADA, Time in Range (TIR), which is the percentage of time blood glucose levels remain within the target range (70–180 mg/dL), not only correlates with HbA1C but also can help predict the risk of complications such as microvascular impairment [26]. Based on these guidelines, uncontrolled diabetes, a complex and clinically-relevant phenotype, is defined as a condition where both blood glucose levels (TIR: 70–180 mg/dL) and HbA1C levels (>7.0%) remain above the target range despite the use of multiple antidiabetic medications. Therefore, the definition of uncontrolled diabetes is closely tied to TIR, HbA1C levels, and the number of antidiabetic medications used, rather than solely focusing on glycemic status.

## 3. Insight into the Monogenic and Polygenic Causes of Uncontrolled T2DM

T2DM is a polygenic disorder [27,28], with multiple genetic risk alleles contributing to an individual’s susceptibility [29]. Research to date suggests that periodic reevaluation of genetic variants associated with diabetes has led to convincing replications of associations with T2DM [30]. While hundreds of genomic sites have been linked to an increased risk of T2DM, only a small fraction are located within protein-coding regions of DNA. Interestingly, many of the polymorphisms identified through genome-wide association studies (GWAS) are found in noncoding regions, which often play a crucial role in the development of human diseases [31].

Diabetes is a multifactorial disease characterized by significant interindividual variation in terms of genetic makeup, etiology, health, lifestyle, age of onset, and disease penetrance. It is prevalent due to the combined influence of genetic and environmental factors, imposing substantial health and societal burdens. Consequently, diabetes presents a significant challenge in terms of both research and management. A key area of focus in diabetes research is the understanding of gene-environment interactions that destabilize glucose homeostasis, increasing disease risk. In this section, we will highlight the most common disease-causing variants related to maturity-onset diabetes of the young (MODY) and uncontrolled T2DM, and explore the molecular, cellular defects, and physiological abnormalities associated with these variants.

### 3.1. The Complex Relationship Between MODY, T2DM, and Genomic Instability

**Figure 3:**
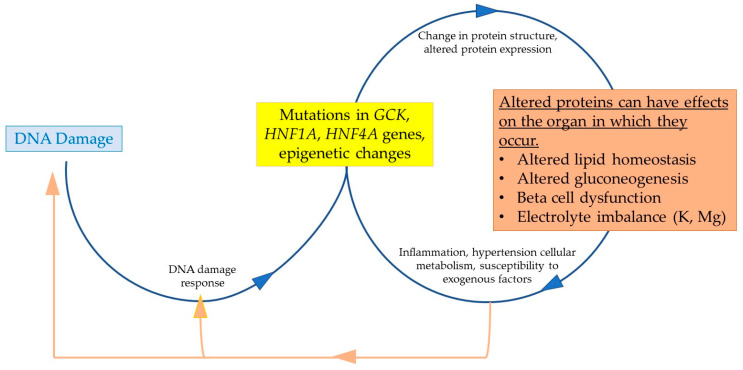
Diagram describing the relationship between metabolic processes and DNA damage and how they contribute to MODY or T2DM.

MODY, T2DM, and genomic instability are intricately linked (Figure 3). DNA instability leads to mutations or epigenetic changes through errors in DNA replication or the replication of previously damaged DNA strands. These alterations can change protein structure and expression, potentially destabilizing glucose homeostasis. The repair of DNA damage is tightly regulated by a network of DNA repair pathways. When genetic information is altered, these changes can be replicated and passed down to subsequent generations through gene flow within populations. The complex relationship between DNA damage and repair can easily become dysregulated, contributing to diseases like MODY or T2DM.

T2DM, in particular, exhibits a marked increase in DNA damage and a decrease in DNA repair capacity due to factors such as hypertension, chronic inflammation, metabolic dysfunction, and susceptibility to environmental stressors. These factors contribute to cellular damage, which in turn affects glucose regulation and exacerbates the disease.

### 3.2. Genetic Contributions to Diabetes: MODY and Uncontrolled T2DM

Diabetes is a complex trait influenced by numerous genes, with variations in genes related to insulin production and secretion, ion channel function, and glucose metabolism being central to disease development. Recent research has shed light on how specific genetic variants influence diabetes susceptibility, offering new insights into its etiology and potential therapeutic targets.

This section focuses on two key aspects: (i) the genes most commonly associated with rare variants causing MODY across different populations, and (ii) the genes identified as harboring rare variants that increase the risk of developing uncontrolled T2DM. We will explore current knowledge regarding the role of these genetic variants in diabetes, discuss the general aspects of glucose metabolism and regulation controlled by these variants, and consider their impact on metabolic processes. Additionally, we will highlight the potential opportunities for personalized medicine based on genetic insights.

This review focuses on specific gene variants such as *PPARγ*, *TCF7L2* (common intronic rs7903146 polymorphism)***,*** *KCNJ11*, *GLIS3*, *UCP1*, *OPG*, and *LEP*, as these variants represent strong candidates due to their roles in insulin regulation and signaling. These genes are well-supported by multiple GWAS and play significant roles in pancreatic beta-cell function and energy metabolism within the context of T2DM pathogenesis (Table 1). Additionally, these genes encode proteins that have recently emerged as therapeutic targets for the development of new treatments for uncontrolled diabetes.

### 3.3. The Candidate Genes Associated with the Development of MODY

While the majority of individuals with T2DM have a polygenic disorder involving multiple genes across different chromosomes, MODY (maturity-onset diabetes of the young) is a monogenic form of diabetes that primarily affects adolescents and young adults. It is characterized by refractory diabetes, a positive family history, and poor glycemic control. MODY results from defects in pancreatic islet cell development, which impair insulin secretion. Over 50% of MODY cases are misclassified as type 1 diabetes mellitus (T1DM) or T2DM [61]. Individuals with MODY often exhibit characteristics that do not align with the typical presentations of either T1DM or T2DM, presenting a diagnostic challenge. Notably, there is evidence suggesting that a small percentage of patients diagnosed with T2DM may actually have MODY [62].

While T2DM generally affects 30–40% of first-degree relatives, MODY exhibits high penetrance, with most carriers showing signs of diabetes within three or more generations [63]. The pathophysiologic characteristics of individuals with MODY include a lack of significant obesity, normal triglyceride levels, mild hyperglycemia, and no signs of insulin resistance or metabolic syndrome. Measurable C-peptide levels also help in diagnosing MODY. Patients with MODY typically test negative for autoantibodies (such as islet cell antibody, insulin autoantibody, insulinoma-associated protein-2 autoantibody, zinc transporter autoantibody, and glutamic acid decarboxylase antibody) [64]. In particular, a negative glutamic acid decarboxylase (GAD) antibody at the time of diabetes diagnosis is an important predictive factor in distinguishing MODY from T1DM [65].

Numerous independent studies have identified rare variants in genes such as *HNF4A*, *GCK*, *HNF1A*, *PDX1*, *HNF1B*, *NEUROD1*, *KLF11*, *CEL*, *PAX4*, *INS*, *BLK*, *ABCC8*, *KCNJ11*, and *APPL1* that are associated with MODY and may influence disease through multiple mechanisms [66]. These genes are involved in various functions, including the development and function of the kidneys, liver, pancreas, and genitourinary tract, regulation of pancreatic beta-cell function, digestion of dietary fats (specifically cholesterol ester hydrolysis), and the development, differentiation, and signaling of insulin-producing beta cells. They also regulate insulin secretion, potassium ion transport, and cellular functions, as well as insulin and adiponectin signaling pathways.

However, the main pathway emerging from the collective study of these genes suggests that most of them are involved in β-cell glucose sensing and glucose metabolism in beta cells, or the activation of ATP-dependent potassium channels [67]. While other genes with rare variants have been proposed as being involved in MODY, these associations have not yet been replicated in independent studies and are, therefore, not discussed in detail here. The most common mutations associated with MODY involve glucokinase (Gck) (MODY 2), hepatic nuclear factor 1-alpha (Hnf1α) (MODY 3), and hepatic nuclear factor 4-alpha (Hnf4α) (MODY 1), which together account for approximately 94% of MODY cases [67,68,69]. Given the significant contribution of these three genes to MODY, we will focus on relevant points regarding their mutations and clinical phenotypic abnormalities.

#### 3.3.1. Genes Harboring Rare Variants That Cause GCA-MODY

Glucokinase (GCK) is a rate-limiting enzyme in the glycolysis pathway that converts glucose to glucose-6-phosphate [70]. As the glucose sensor in pancreatic beta cells, GCK plays a crucial role in glucose homeostasis. Upon activation by increased glucose levels, GCK catalyzes the conversion of glucose to glucose-6-phosphate in both the liver and pancreatic beta cells, a process that ultimately leads to the production of ATP. The elevated ATP levels close ATP-sensitive K^+^ channels, causing voltage-gated Ca^2+^ channels to open and allowing the influx of calcium ions. This influx of calcium triggers insulin secretion.

Mutations that impair the function of GCK can either inactivate the enzyme or make it overactive. Inactivating mutations result in reduced GCK activity, leading to decreased insulin secretion. On the other hand, activating mutations cause GCK to become overactive, leading to uncontrolled insulin secretion and hypoglycemia. Over the past two decades, studies have focused on the molecular, cellular, and physiological consequences of inactivating *Gck* gene mutations. These mutations are characterized by reduced insulin secretion and a higher glucose threshold needed to stimulate insulin release. Inactivating *Gck* gene mutations are implicated in various monogenic diabetes disorders, such as permanent neonatal diabetes mellitus (PNDM) and MODY [71].

Inactivating mutations in GCK can be classified into two categories: (1) homozygous inactivating mutations in the *Gck* gene, which lead to severe conditions like PNDM, and (2) heterozygous inactivating mutations in the *Gck* gene, which result in a milder form of the condition, such as GCK MODY, typically exhibiting fasting hyperglycemia [72]. For further details on the structure of GCK and its role in the development of diabetes, refer to other sources [73].

GCK variants are recognized as a genetic risk factor for MODY in many populations [74], with GCK-MODY having an estimated population prevalence of 0.11–0.21% [75]. Notably, individuals with pathogenic glucose-elevating GCK variants and a T2D diagnosis have nearly three times the T2D-associated complications compared to population controls [69]. MODY affects approximately 1 in 10,000 Caucasian adults [76], but GCK-MODY prevalence is about 1 in 1000 individuals [69,77]. In non-obese Maltese adults with diabetes and prediabetes, GCK-MODY prevalence reaches nearly 2%, highlighting the importance of molecular testing in young adults with diabetes [78]. This suggests that GCK-MODY is more likely to occur in certain geographic regions due to epigenetic mechanisms that influence gene expression.

Patients with heterozygous inactivating GCK variants typically exhibit slightly elevated blood sugar levels [33] but remain asymptomatic. Recent studies have replicated the genetic association of GCK variants with MODY in patient cohorts and assessed the functional consequences of various polymorphisms by comparing FGM (flash glucose monitoring) and HbA1c levels between individuals with the protective GCK genotype and those with the disease-associated genotype [34]. The study found that pathogenic (P) or likely pathogenic (LP) variants, such as C220Y and G72R, are consistent with causal GCK variants for MODY [34]. This suggests that modulation of GCK activity may represent a potential therapeutic approach for treating GCK variant-induced MODY.

The functional effects of GCK-MODY variants on disease pathogenesis remain largely unclear, and approximately 80% of patients with GCK variant-induced MODY remain undiagnosed due to symptom similarities with other types of diabetes [79]. As a result, many individuals with GCK mutations are incorrectly treated with insulin injections. To aid in diagnosis, the Exeter MODY probability calculator, a validated mathematical model, has been developed to predict the likelihood of a patient having MODY based on clinical features such as HbA1c, beta-cell autoantibodies, plasma C-peptide levels, family history of diabetes, insulin/non-insulin hypoglycemic agent use, BMI, and age at diagnosis [80].

The calculator has demonstrated good discrimination between MODY and T1DM or T2DM in European adults [81], Portuguese [80], and Chinese [82] populations. However, it struggles to differentiate MODY from T2DM in an ancestrally diverse pediatric population [81,83]. Therefore, the MODY probability calculator, in combination with gene-specific biomarkers, can be a valuable tool for diagnosing MODY and distinguishing it from other forms of diabetes.

#### 3.3.2. An Update on HNF1A

The hepatic nuclear factor (HNF) and HNF3 subfamily are a group of proteins involved in various biological processes [84]. Diseases associated with mutations in HNF1α are genetic conditions that impact a wide range of physiological functions, such as lipid homeostasis, gluconeogenesis, and ureagenesis. These mutations are linked to conditions like MODY (maturity-onset diabetes of the young), renal abnormalities (including hyperuricemia, electrolyte disturbances, and polyuria), and defects in beta-cell insulin secretion [85,86], which contribute to the pathogenesis of diabetes and its chronic complications.

HNF1α (also known as HNF3) is the most common genetic cause of MODY in adult populations [87]. Interestingly, some mutations in HNF1α do not lead to MODY3 but rather increase susceptibility to T2DM [87]. While the clinical presentation of HNF1-MODY varies, most patients with HNF1α-MODY3 show elevated postprandial blood glucose without ketosis [88]. Evidence suggests that blood glucose control is crucial for these individuals, as uncontrolled blood sugar can lead to diabetic complications such as vascular damage in the eyes and kidneys. Studies have also shown that individuals with mutations in the Hnf1α gene have an increased risk of cardiovascular mortality compared to unaffected family members [89].

HNF1α-MODY typically manifests in adolescence or early adulthood, though some individuals are diagnosed later in life. Identifying mutations in Hnf1α gene is important for diagnosing MODY and understanding its mechanisms, which can guide the development of targeted molecular therapies for precision medicine.

Mutations in HNF1α protein may disrupt gene expression pathways in tissues such as the pancreas, liver, and kidneys, leading to abnormal blood glucose regulation and linking HNF1α dysfunction to both MODY and T2DM risk [84].

In the pancreas, HNF1α plays a crucial role in pancreatic development and in maintaining the function of both alpha and beta cells in primary human islets [90]. Insufficient HNF1α leads to progressive reductions in insulin secretion over time [35]. Heterozygous mutations in the *Hnf1α* gene result in MODY3 [38]. Impaired beta-cell glucose sensing is a hallmark of HNF1α deficiency, and knockout of the *Hnf1a* gene in mice leads to impaired insulin release and disrupted aerobic glucose metabolism [91]. This suggests that HNF1α deficiency in humans impairs aerobic glycolysis and mitochondrial function [91,92].

In the liver, the *Hnf1α* gene regulates the expression of genes involved in liver development, function, and metabolism, including those associated with glucose homeostasis and lipid metabolism [93,94,95]. Mutations in *Hnf1α* can lead to altered serum lipoprotein levels [37,96]. These mutations also promote fat accumulation in the liver, while reduced HNF1α expression contributes to liver steatosis [97]. The most common mutation in MODY3 patients is P291fsinsC-HNF1A [98], which causes both liver steatosis and beta-cell dysfunction [38,93]. Recent studies show that this mutation induces the complement factor D (CFD) pathway, triggering liver steatosis and inflammation [95]. HNF1α knockout mice also exhibit hepatomegaly and fatty liver [99], and homozygous Hnf1α deficiency in mice leads to hyperbileacidemia, hypercholesterolemia, and altered bile acid synthesis and uptake [100]. However, heterozygous mutations in HNF1α do not seem to cause significant lipid metabolism abnormalities [94], although they are associated with lipotoxicity via reduced fatty acid-binding protein 1 (FABP1) expression [97]. Thus, individuals with HNF1α mutations may develop liver steatosis [95], further implicating HNF1α as a critical regulator of key enzymes involved in gluconeogenesis.

Certain variants of HNF1α are associated with an increased risk of T2DM. Variants such as E508K, I27L, and A98V have been linked to T2DM susceptibility [101,102]. Furthermore, polymorphisms I27L and A98V are also associated with HDL cholesterol levels. Interestingly, the rs2637248 polymorphism in the LRMDA gene correlates with the age of diabetes onset in individuals with HNF1A mutations [103]. Impaired HNF1α function can also influence LDL-cholesterol and C-reactive protein levels [104,105].

In the kidneys, HNF1α binds to several sites on megalin and cubilin, which are involved in endocytosis [106]. In Hnf1α knockout mice, this leads to a significant reduction in the uptake of β2-microglobulin, indicating impaired endocytosis. Although no convincing *Hnf1α* gene variant-induced abnormal endocytosis has been identified in humans, individuals with HNF1α mutations exhibit low molecular weight proteinuria compared to individuals with non-MODY HNF1A diabetes [106]. Additionally, HNF1α is involved in glucose reabsorption from the glomerular filtrate in both humans and mice [107]. HNF1α deficiency reduces the expression of sodium-glucose co-transporter-2 (SGLT2) [108]. As a result, HNF1α-MODY patients may exhibit glycosuria and altered endogenous glucose production. Reduced SGLT2 expression impairs glucose reabsorption in the kidney tubules, leading to higher concentrations of glucose in the urine even when blood glucose levels remain normal. This glycosuria often occurs before any decline in beta-cell insulin secretion, typically years before MODY3 diagnosis [109]. Thus, mutations in HNF1α lead to proximal tubular dysfunction in the kidneys.

HNF1α is a strong regulator of organic cation transporter 1 (OCT1) expression in the liver [110,111,112]. However, the exact mechanism through which HNF1α regulates OCT1 expression is still unclear. Given that metformin, a first-line therapy for diabetes, is transported into the liver by OCT1 [113], it is possible that HNF1α genetic variations could influence metformin bioavailability and drug elimination. OCT1-mediated transport of metformin is essential for its therapeutic effect, and variations in the OCT1 gene (such as the OCT1-420del variant) result in reduced metformin transport activity [113]. As such, HNF1α genetic mutations could potentially alter OCT1 expression, thereby influencing the effectiveness of metformin therapy and affecting its pharmacokinetics [114,115].

In conclusion, HNF1α is a critical regulator of multiple processes in the pancreas, liver, kidneys, and other tissues, with mutations leading to a wide range of metabolic disorders, including MODY3. Understanding the pathophysiology of HNF1α dysfunction offers valuable insights into precision medicine and targeted therapies for individuals with these genetic mutations.

#### 3.3.3. HNF4A-MODY Overview

Similar to HNF1A-MODY, patients with HNF4A-MODY typically exhibit signs and symptoms of diabetes that develop gradually during childhood or young adulthood. Clinical manifestations include abnormal conditions such as elevated blood glucose levels, frequent polyuria, and excessive thirst. If left uncontrolled, prolonged high blood glucose levels can result in serious health complications, such as nephropathy and retinopathy, due to damage to small blood vessels in the kidneys and eyes, respectively. The oral glucose tolerance test (OGTT) is often considered a more reliable diagnostic tool than fasting blood glucose (FBG) for patients with mutations in *HNF1A* and *HNF4A* [116,117].

Both HNF1A-MODY and HNF4A-MODY are progressive conditions, with penetrance increasing with age. A higher proportion of mutations are observed in exons two and four of *HNF1A*, and in exons seven and eight of *HNF4A*. Specific mutations in these genes are frequently found in certain populations due to a founder effect [118]. The prevalence of *HNF1A* mutations is higher compared to those in *HNF4A*. In contrast to HNF1A-MODY, babies with HNF4A-MODY are more likely to have higher-than-average birth weights or experience severe hypoglycemia at birth. These factors can help differentiate between MODY 1, MODY 3, and T2DM. Pathogenic variants in *HNF4A* are commonly associated with MODY [119]. Additionally, epigenetic modifications of HNF4A-MODY increase the likelihood of developing hepatocellular adenomas [120].

HNF4A protein is expressed in various adult tissues, including the pancreas, gut, liver, and kidneys [121], and defects in HNF4A function can result in diabetes. HNF4A is especially highly expressed in hepatoblasts [84], but the pancreas appears to be primarily affected in patients with HNF4A-MODY [122]. The pathophysiological mechanisms of HNF4A-related diabetes resemble those of mutations in the Hnf1α gene [123]. Mutations in the *Hnf4α* gene are associated with an increased risk of developing T2DM [124]. The HNF4α protein plays a central role in regulating lipid homeostasis, glycolytic enzymes, and glucose transporters, which suggests that it plays a crucial role in metabolic control [125,126].

HNF4A undergoes extensive post-translational modifications, enhancing its diversity and regulatory capabilities [127]. The gene produces multiple isoforms under the control of two distinct promoters, P1 and P2, which are expressed in the epididymis and intestine in humans [128,129]. HNF4A is associated with intestinal lipid metabolism, although this function remains somewhat unclear due to redundancy with HNF4G [127,130].

HNF4a is a regulator of numerous gene expression across multiple organs [127]. HNF4A is one of the primary transcriptors that significantly regulates the release of glucose-dependent insulinotropic polypeptide (GIP) in response to fat intake [124], with studies showing that conditional deletion of HNF4α in the intestine reduces GIP levels. Mutations in the Hnf4*α* gene also impair transcriptional activity and insulin secretion [40].

Mutations in both *Hnf1α* and *Hnf4α* genes contribute to beta-cell dysfunction [131]. These impaired beta-cells lead to hyperglycemia, which can result in diabetic angiopathy. Patients with HNF4A mutations often show reduced glucose-stimulated insulin secretion and hypoinsulinemia-induced hyperglycemia, but without evidence of beta-cell autoimmunity [63], suggesting a specific dysfunction in beta-cell regulation. Patients with HNF4A-MODY also exhibit lower circulating triglyceride levels [132]. If blood glucose levels are poorly controlled, the risk of both microvascular and macrovascular complications is significantly elevated [63].

Recent findings from Turkey’s largest genetically diagnosed series show that HNF4A-MODY, while rarer than HNF1A-MODY (which occurs in about 19% of cases), is still a relevant diagnosis [133]. Similarly, HNF1A-MODY is more commonly found than HNF4A-MODY in European countries [76] and the USA [134]. In South India, HNF1A-MODY is reported more frequently than HNF4A-MODY [135]. Notably, the prevalence of retinopathy and nephropathy in patients with HNF4A-MODY is higher than in those with T2DM [135]. While GCK-MODY is the most common type in Japan [136], the proportion of patients with GCK-MODY is similarly higher than patients with HNF-MODY in other countries’ registries. More detailed information about the most commonly reported mutations in HNF4A are summarized in (Table 2).

A study from the UK Biobank provides robust statistical evidence supporting the fact that mutations in HNF1A, HNF4A, GCK, and KCNJ11 are among the most common well-established forms of MODY [137]. The most frequently reported mutation in HNF4A is p.R114W (previously p.R127W [138]), which is heterozygous in nature [139]. This mutation is associated with an early onset of diabetes, with 54% of patients developing the condition by age 30. These patients also show reduced sensitivity to sulfonylurea therapy. A study from Turkey identified a novel Hnf4α gene variant (c.110T>C) that was deemed disease-causing by MutationTaster, in addition to the previously reported variant (c.1097C>G) [41].

HNF4A-MODY should be considered a potential diagnosis in young diabetic patients, and genetic testing is essential for establishing an appropriate therapeutic approach.

#### 3.3.4. KCNJ11 Variants That Are Associated with an Increased Risk of T2DM in Adults

*KCNJ11* is a member of the potassium channel genes [43]. The protein encoded by *KCNJ11* is an inward-rectifying channel (Kir6.2, the pore-forming subunit of the β-cell potassium channel), which is characterized by allowing the efflux of potassium into the cell [140]. The elevation of cytosolic potassium levels depolarizes the cell membrane and subsequently activates the calcium ion channel, leading to the elevation of calcium levels. Increased intracellular free calcium levels are essential for insulin secretion from pancreatic β-cells [141]. These calcium channels trigger the voltage-dependent potassium channels to repolarize the cell membrane. Kir6.2 protein (consists of 4 subunits) is controlled by G-protein and is associated with the sulfonylurea receptor 1 (made of 4 subunits, regulatory subunits) encoded by the ABCC8 gene [142]. Kir6.2 is implicated in the regulation of cellular metabolism and contributes to the mechanisms by which hypoglycemia stimulates glucagon release from pancreatic α-cells. Several reviews can provide the readers with a more detailed understanding of the structure, function, and regulation of potassium inward-rectifying channel superfamily [43,140,143].

Studies strongly indicate that mutations in KCNJ11 increase autosomal dominant non-insulin-dependent diabetes mellitus type 2 risk in numerous ethnic groups such as Caucasian and in some Ascian populations, in addition to causing both transient neonatal diabetes and permanent neonatal diabetes [43,45,144]. Mutations in KCNJ11 typically occur de novo during gametogenesis or embryogenesis [145]. While genome-wide association studies on the *Kcnj11* gene have identified over 180 single nucleotide polymorphisms (SNPs) [146,147], three of which are located in the coding regions and are associated with an increased risk of diabetes [43]. These three SNPs include rs5215 (I337V), rs5218 (A190A), and rs5219 (E23K) [44], which will be discussed here.

*Kcnj11* rs5218, a synonymous variant, is associated with the pathogenesis of T2DM, whereas rs5215 and rs5219, non-synonymous variants, are found in individuals with T1DM and T2DM. The *Kcnj11* gene variant (rs5219) is strongly related to the levels of the circulating HbA1c in this disease [148] and can cause transient neonatal diabetes or permanent neonatal diabetes. Both rs5215 and rs5219 polymorphism were associated with blood pressure levels among patients with T2DM [149,150,151]. The correlation between rs5219 in patients with T2DM and the medication response is controversial. *Kcnj11* rs5219 gene polymorphism also is found to be implicated in gestational diabetes mellitus in people in China [152] but not in individuals from Sweden [153,154]. These studies suggest that background polygenic risk in the population in China differs greatly with respect to disease prevalence and incidence. A meta-analysis showed that the *Kcnj11* rs5219 polymorphism is a risk factor for developing T2DM in Caucasians and in populations from East Asia [155], and an independent predictor of T2DM in the Iraqi population in the Middle East [156]. Another study provides no evidence supporting a relationship between rs5219 and the risk of diabetes in Iranian [157]. The lack of association could potentially be due to the absence of other environmental elements such as obesity [158] and hypertension [149]. Rs5219 is found to be associated with several diseases including hyperinsulinemic hypoglycemia [159], familial 2 [146], and MODY 13.

The role of rs5219 in medication response remains contentious. While the *Kcnj11* gene polymorphism (rs5219) is associated with hepatitis insulin sensitivity [160], it is not associated with diabetic peripheral neuropathy [161]. Several studies provide evidence that diabetic patients with the *Kcnj11* rs5219 variant are susceptible to metformin therapy [162], metformin and sulfonylurea combination therapy [163], metformin and gliclazide therapy [164] in different ethnic groups [162]. Patients with *Kcnj11* gene polymorphism (rs5219) are more responsive to glimepiride and glibenclamide than those being treated with gliclazide [148]. However, another study indicates that patients with rs5219 variant exhibit the impairment of glibenclamide-induced insulin release, highlighting an example of pharmacogenetics in T2DM [165].

These findings highlight the importance of genetic variability in drug response, underscoring the potential for personalized treatment strategies to reduce healthcare disparities and enhance precision medicine.

### 3.4. The Candidate Genes Associated with the Development of Late-Onset T2DM

#### 3.4.1. The Transcription Factor 7-like 2 (TCF7L2) and Its Association with Beta-Cell Dysfunction in T2DM

The prevailing view is that genetic testing for predicting T2DM in high-risk individuals has limited clinical value, due to the small effect size of genetic loci and the low discriminative ability of such tests. However, recent studies indicate that the transcription factor 7-like 2 (TCF7L2, previously known as TCF4) is the most potent genetic locus for T2DM risk [47,166]. TCF7L2 is implicated in approximately one-fifth of all T2DM cases [167]. It binds to DNA via a high-mobility group domain, playing a key role in blood glucose homeostasis [168]. TCF7L2 is also a critical component of the Wnt signaling pathway, which significantly regulates glucose metabolism by influencing insulin production and the function of GLP-1 [169], as well as lipid metabolism [170]. For a deeper understanding of the involvement of the Wnt signaling pathway and TCF7L2 in diabetes, interested readers can refer to [169,171].

The association between the *tcf7l2* rs7903146 gene variant and T2DM susceptibility [4] has been confirmed in several follow-up studies [47]. This finding has been replicated across diverse cohorts, including East Asian, European, and West African populations [172], as well as in the U.S. [168]. Importantly, the T2DM risk associated with the TCF7L2 SNP has been linked to complications of the disease and has been shown to influence the effectiveness of oral hypoglycemic agents, such as sulfonylureas, which stimulate insulin release from the pancreas [166]. Notably, the *tcf7l2* rs290487 gene variant has been associated with elevated fasting blood glucose and HbA1c levels in cirrhotic diabetic patients compared to cirrhotic non-diabetic patients [173]. The majority of T2DM risk variants of TCF7L2 are strongly associated with beta-cell dysfunction [174]. Thus, the *tcf7l2* rs7903146 gene variant is clinically relevant for T2DM prediction.

#### 3.4.2. GLIS3 Is Considered a Contributing Factor to Polygenic Nature of T2DM

Gli-similar 3 (GLIS3), a key transcription factor involved in insulin production, plays a compensatory role in β-cell proliferation in adults [67,175,176]. The protein encoded by GLIS3, a member of the Krüppel-like zinc finger protein subfamily, is expressed in the pancreas, thyroid, and kidney. GLIS3 is also expressed in various stem and progenitor cells in both adults and during mammalian development [177]. Loss of GLIS3 function leads to the suppression of metabolic genes involved in oxidative phosphorylation, fatty acid oxidation, and the tricarboxylic acid cycle [178]. In a spatial transcriptomic study, GLIS3 was found to be upregulated in both fibroblasts and endothelial cells following vein harvesting and implantation, a state associated with inflammation and thrombosis [179]. Previous studies have shown that GLIS3 knockdown accelerates β-cell apoptosis, which is further exacerbated by proinflammatory cytokines or palmitate, potentially contributing to β-cell loss in T2DM [180]. These findings suggest that GLIS3 plays a protective role in the postnatal kidney against cyst formation and in vascular remodeling.

Although mutations in the *glis3* gene are known to cause neonatal diabetes syndrome, GWAS have shown a strong association between GLIS3 and both T1DM and T2DM across multiple populations [181]. Mutations in GLIS3 have been implicated in various pathologies, including neonatal diabetes mellitus, congenital hypothyroidism, congenital glaucoma, and polycystic kidneys [181,182,183]. Defective GLIS3 function leads to impaired insulin production, which may cause abnormal pancreatic development [184], dysfunctional β-cells, or β-cell destruction [183,185,186]. In vitro, GLIS3 deficiency in β-cells activates the intrinsic pathway of apoptosis [180], thereby increasing susceptibility to diabetes.

Recently, evidence strongly indicates that mutations in GLIS3 increases type 2 diabetes risk, in addition to GLIS3 involvement in monogenic diabetes [51]. Sulfonylureas might be used as the first-line therapy to manage T2DM in the carriers. Individuals with pathogenic or likely pathogenic (P/LP) GLIS3 variants are at an increased risk for T2DM [51]. Heterozygous P/LP GLIS3 variants are associated with elevated T2DM risk, with these variants appearing to alter β-cell function. Rare missense mutations in GLIS3 and the glis3 rs7034200 variant have been linked to elevated HbA1c levels, and reduced β-cell mass and function [52,187]. The GLIS3 rs7034200 variant predisposes the Asian population to a higher risk of developing T2DM, with the risk further exacerbated by lifestyle factors such as diet and smoking [52]. Additionally, the *glis3* rs10758593 variant has been significantly associated with both diabetic retinopathy and diabetic nephropathy in an Egyptian population [17]. GLIS3 variants contribute to an increased risk of T2DM. Sulfonylureas may be an effective treatment to reduce blood sugar levels and related complications in carriers of these variants.

#### 3.4.3. Peroxisome Proliferator-Activated Receptor Gamma (PPARγ) in Diabetes, Obesity, and Atherosclerosis

Diabetes arises from the long-term interaction of various internal and external risk factors. Disruption of normal metabolic processes involving proteins, fats, and, especially, carbohydrates increases the risk of diabetes mellitus, dyslipidemia, vascular disease, obesity, and nonalcoholic fatty liver disease (NASH), all of which contribute to glucotoxicity and lipotoxicity [188]. Endothelial dysfunction-induced reductions in insulin signaling lead to insulin resistance, linking the metabolic and cardiovascular components of metabolic syndrome [189].

PPARγ plays a crucial role in adipose tissue, a key player in glucose and lipid homeostasis, and is involved in the prevention of oxidative stress. PPARγ is the molecular target for the antidiabetic effects of thiazolidinediones (TZDs). It improves systemic insulin sensitivity by activating thermogenesis and promoting the release of adipokines from white adipose tissue (WAT) and brown adipose tissue (BAT) [190]. The importance of modulating PPARγ continues to be emphasized in recent research.

PPARγ is a ligand-activated nuclear transcription factor that regulates glucose homeostasis [191] and has a significant effect on lipid metabolism [192]. PPARγ agonists have been shown to reduce fibrosis in organs like the pancreas and liver [193], which are critical in regulating glucose metabolism and maintaining glucose homeostasis. Deficiencies in PPARγ and leptin are associated with metabolic syndrome, characterized by dyslipidemia, renal hypertrophy, and elevated levels of the profibrotic cytokine transforming growth factor beta (TGFβ) in the kidneys [194]. Interestingly, recent preclinical studies indicate that obesity causes a reduction in PPARγ expression in white adipose tissue [195]. Reduced PPARγ levels impair the adipocyte uptake of glutamine and methionine, two epigenetic activators of Bmal1 (a clock gene). This study suggests that PPARγ may facilitate a futile cycle between circadian disruption and obesity development [195].

Notably, studies have shown that deletion of the *pparγ* gene in the epiblast of mice leads to the development of T2DM and renal fibrosis [196]. Similarly, macrophage-specific deletion of PPARγ results in lupus-like autoimmune glomerulonephritis [197], highlighting the critical role of PPARγ in inflammation. Inflammation, reactive oxygen species, and diabetes have a bi-directional relationship [198].

Several studies have extensively examined the role of PPARγ in metabolic disorders [196,199,200,201]. Disease-associated mutations in PPARγ can result in either complete or partial loss of function [54], depending on their impact on the residual activity of the encoded mutant PPARγ. Variants in the *pparγ* gene have been linked to severe obesity [201], insulin resistance [54], a decreased risk of T2DM in certain ancestries [202], or an increased risk of T2DM [54], hypercholesterolemia [203], and systemic sclerosis characterized by diffuse fibrosis and vascular abnormalities [204].

TZDs, a class of full PPARγ agonists, increase insulin sensitivity in peripheral tissues and help reduce hyperglycemia [205]. TZDs have been reported to have protective effects on vascular endothelium in patients with T2DM and coronary atherosclerosis [206]. However, TZDs can cause edema, which may increase the risk of heart failure, a common complication of T2DM. A recent report indicates that TZDs may increase the risk of cardiovascular diseases, such as myocardial infarction and heart failure [207]. Interestingly, lobeglitazone, a novel TZD, decreases the risk of cardiovascular complications without increasing the risk of heart failure [208].

Despite these concerns, TZDs remain notable in large cohorts of US outpatients with T2DM. It has been suggested that targeting TZDs to patients with T2DM who are at low risk for heart failure is important [209]. The prevalence of TZD-induced edema is higher in predominantly Caucasian populations than in Asian populations [210]. Over the past two decades, several genome-wide association studies (GWAS) have revealed numerous SNPs in the *pparγ* gene. Variants like rs4684847 and rs1801282 are susceptible variants for T2DM [53,54,55]. The PPARγ rs4684847 gene variant is significantly associated with lipoprotein(a) [211], baseline body mass, and hypertension [212]. Notably, significant differences in susceptibility to *pparγ* gene variants have been observed across different ethnic groups. Evidence shows that the pparγ rs4684847 gene variant does not predispose South Indian patients to diabetic dyslipidemia [213].

Although identifying the patients with PPARγ variants who are ideal candidates for TZDs is not yet feasible, studies have shown that genetic defects in metabolism can be rescued by environmental (rosiglitazone)-driven epigenomic modulations [214]. According to these studies, the upregulation of PPARγ is a potential therapeutic pathway for managing diabetes. In conclusion, avoiding the use of TZDs in patients with heart failure is recommended. These studies provide irrefutable evidence that certain common PPARγ variants could contribute to the pathogenesis of T2DM, particularly in relation to diabetes-related complications across different ethnic groups.

#### 3.4.4. The LEP Variant and Its Potential Contribution to Insulin Resistance and Metabolic Syndrome

Diets high in fat, carbohydrates, and fructose, and low in protein, have been shown to reduce leptin secretion, contributing to a state known as leptin resistance [215]. In contrast, a high-protein diet can lead to a sustained reduction in appetite and a decrease in plasma leptin levels [216]. Leptin levels are positively correlated with body mass index (BMI) and fat mass [217]. It is primarily produced by white adipose tissue and the deficiency of *LEP* causes insulin resistance [218]. Congenital leptin deficiency is associated with excessive hunger and weight gain, while elevated leptin levels have been linked to T2DM [219]. It is suggested that subcutaneous leptin therapy is beneficial in some severe lipodystrophy in monogeneic-resistant diabetes [220]. Interestingly, recombinant leptin treatment in children with leptin deficiency has resulted in sustained body weight reduction, particularly through fat loss [221].

Although the genetic causes of T2DM are complex, certain rare genetic variants have been identified that increase the risk of developing the disease. A recent study highlighted a rare enhancer variant near the *lep* gene (rs147287548) [42] and the LEP G2548A polymorphism [56]. These variants are particularly prevalent in African and African American populations, as well as in the Malaysian population, and significantly increase the risk of T2DM [42]. The *lep* gene encodes leptin, a hormone primarily produced by white adipose tissue, and its deficiency has been shown to contribute to insulin resistance [218].

Furthermore, NFATc transcription factors, which are modulated in obesity, play a crucial role in regulating glucose and insulin homeostasis [222]. The *lep* rs147287548 polymorphism has been shown to disrupt the NFATc motif, leading to altered apolipoprotein A levels and reduced high-density lipoprotein (HDL) cholesterol levels in individuals without diabetes [42]. The identification of this mutation in LEP helps guide the management of T2DM.

#### 3.4.5. The UCP1 rs45539933 Gene Variant Is Associated with Obesity and Diabetes

Patients with T2DM often develop microvascular complications such as retinopathy, neuropathy, and nephropathy—serious and progressive conditions that are common in T2DM [223]. These microvascular diseases can lead to macrovascular complications, affecting arteries and veins, which significantly impact patients’ morbidity and mortality [224,225]. Chronic elevated blood glucose levels play a crucial role in initiating diabetic vascular complications through mechanisms such as the formation of advanced glycation end products, abnormal activation of the renin-angiotensin system, and increased production of reactive oxygen species.

Uncoupling protein 1 (UCP1) is inversely related to insulin resistance [226]. It is involved in oxidative phosphorylation, regulation of energy expenditure, and reduction of oxidative stress [227,228]. These processes are disrupted in metabolic disorders like obesity and diabetes [229]. There is strong evidence that UCP2, a protein found in pancreatic beta-cells, negatively regulates insulin secretion [230]. Inhibiting UCP2 restores glucose-induced ATP production [231] and suppresses insulin secretion [230]. Thus, both UCP1 and UCP2 play significant roles in regulating local tissue homeostasis.

Mitochondrial alterations are evident in brown adipose tissue (BAT) from UCP1 knockout (KO) mice exposed to low environmental temperatures [232]. Studies indicate that UCP1 is dispensable for cold-stimulated thermogenesis [233]. Transcriptional repression of enhancer regions significantly reduces UCP1 protein expression and impairs mitochondrial function in brown adipocytes [234]. However, such repression of UCP1 expression is not typically observed in conditions like obesity and diabetes. Activation of BAT is impaired in healthy individuals carrying specific ucp1 gene variants [235]. Among the most commonly reported variants associated with obesity and diabetes is the *ucp1* rs45539933 variant in the codon region [57,58,59]. In summary, UCP1 and UCP2 have distinct roles in the regulation of mitochondrial-induced oxidative stress, a pathogenic factor in diabetes. The identification of the *ucp1* rs45539933 variant suggests that a polygenic background can influence the penetrance of pathogenic mutation related to obesity and lipid disorders.

#### 3.4.6. The OPG Variant and Its Potential Contribution to Hypertension, and Diabetes

**Figure 4:**
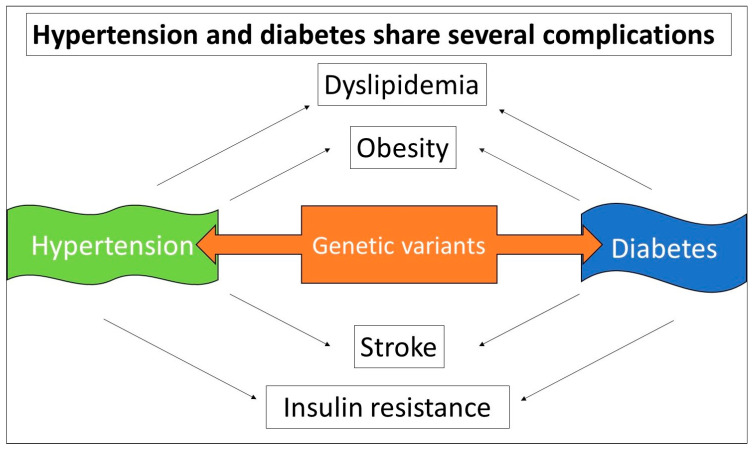
Diabetes and hypertension and their common complications appear to be due to a complex interaction between genetic predisposition and epigenetic factors.

Recent genetic evidence indicates that monogenic rare variants and SNPs are responsible for susceptibility to high blood pressure via alterations in the components of the renin-angiotensin system, including natriuretic peptides, the sympathetic neuronal system, endothelial dysfunction, and inflammation [236,237]. Notably, 50% of people with insulin resistance are susceptible to developing hypertension, in the early phase of the disease [238,239]. This suggests that insulin resistance leads to genetic and epigenetic perturbations that result in hypertension. Both diabetes and hypertension are recognized as the major risk factors for CV disease [240], and have substantial overlap in the CV disease complications, including atherosclerosis, dyslipidemia, and obesity (Figure 4). Needless to say, the prevalence of diabetes-associated complications varies among different ethnic populations [241], leading to the notion that disparities in the variant allele frequencies among different ethnic regions may play a role in disease susceptibility [242].

Vascular calcification, an age-related phenomenon, is common in both diabetes [243] and hypertension [244]. The intimal calcification not only contributes to a reduction in vascular compliance [244] but also is linked to vessel stiffness. Arterial stiffening, a hallmark of atherosclerosis, is a known pathophysiological mechanism of hypertension [245]. Although the underlying mechanisms of diabetes-induced atherosclerosis remain a dilemma, higher levels of arterial calcification are observed in patients with diabetes at the clinical and pathological level [246]. Osteoprotegerin (OPG), a biomarker of vascular calcification, is a decoy receptor for receptor-activator for NF-κB ligand (RANKL), which has been implicated in the pathophysiology of vascular calcification [243]. OPG is also found to be associated with myocardial stiffness [247], hypertension, and diabetes [60,248,249]. Recent genetic evidence indicates that CV risks are higher in T2DM patients with hypertension expressing opg rs2073618 gene polymorphism [60] (Table 1).

In conclusion, the genes discussed above are sufficient on their own to contribute to the development of T2DM. However, genetic linkage analyses have not yet identified GLIS3, PPARγ, LEP, UCP1, and OPG as major contributors to the cause of late-onset T2DM in various populations.

## 4. Diabetes Inheritance and Personalized Treatment Approaches

### 4.1. Current Treatment Guidelines for Diabetes

The ADA recommends a stepwise approach to managing diabetes, with metformin as the first-line treatment, followed by other agents such as sulfonylureas, GLP-1 receptor agonists, or SGLT2 inhibitors (Figure 2). These guidelines emphasize the importance of individual patient factors, including cardiovascular (CV), kidney, and liver health, as well as comorbidities, treatment preferences, and the risk of hypoglycemia [250].

However, emerging data suggest that this “one-size-fits-all” approach may not be effective for all patients. Diabetes is a multifactorial disease, involving endothelial dysfunction, inflammation, oxidative stress, insulin resistance, and coagulation impairments induced by chronic hyperglycemia [251,252]. These factors contribute to thrombotic diseases in T2DM patients [198,253,254]. Moreover, genetic variability, particularly across different ethnic groups, can significantly impact drug efficacy, often leading to suboptimal treatment outcomes.

### 4.2. Genetic Variability and Ethnic Differences in Drug Response

Genetic differences play a crucial role in individual responses to common antidiabetic medications [255]. For example, the *pparγ* gene, a susceptibility locus for T2DM [256], is also the molecular target of TZDs [257]. Similarly, *Kcnj11*, which encodes a potassium channel involved in insulin secretion, is a target for sulfonylureas, with genetic variation influencing medication responses, such as changes in fasting glucose and HbA1c levels [257]. Furthermore, TCF7L2 polymorphisms have been associated with insulin secretion and response to GLP-1 receptor agonists and sulfonylureas [257,258].

Additionally, the *slc2a2* gene encodes the facilitated glucose transporter GLUT2, variations influence hepatic metformin uptake, influencing its therapeutic effectiveness [259]. It is suggested that *slc2a2* rs8192675 can serve as a potential biomarker for stratified medicine [259]. Research also shows that African Americans exhibit a stronger glycemic response to metformin compared to European Americans [260].

These findings carry significant implications. Integrating pharmacogenomic testing into diabetes care could lead to more personalized treatment strategies that maximize efficacy and minimize adverse effects. Genotype-guided treatment protocols, supported by evidence from large-scale, diverse studies, could transform diabetes care into a precision medicine framework. This approach has the potential to not only improve individual outcomes but also address disparities in treatment effectiveness across different population groups, fostering equity in healthcare delivery.

## 5. Obesity and Its Association with Insulin Resistance

Obesity is characterized by excessive body fat accumulation, which poses significant health risks, including reduced life expectancy and a higher incidence of various diseases [261]. The most common measure of obesity is BMI, classifying individuals based on weight relative to height. According to the WHO, a BMI of 25 or higher is classified as overweight, while a BMI of 30 or higher is considered obese. The prevalence of overweight and obesity has steadily increased in both children and adults from 1990 to 2022 [262].

Several factors contribute to obesity, including the consumption of energy-dense, nutrient-poor foods, poor eating habits, lack of physical activity, hormonal imbalances, genetic predisposition, and certain illnesses and medications [263,264]. As a highly complex condition, obesity involves multiple genetic loci and pathways that regulate energy balance, appetite, and metabolism. Among the most prominent complications of obesity are metabolic syndrome, stroke, osteoarthritis, and certain types of cancer (such as endometrial, colon, and gall bladder cancer) [265,266]. Unfortunately, most current treatments for obesity, including pharmacological agents, are associated with serious side effects and variable clinical outcomes [267].

Obesity is closely associated with insulin resistance [268], making it a significant risk factor for the development of T2DM. Genetic studies have identified several genes linked to both obesity and insulin resistance, such as Pro12Ala PPAR-γ, fat mass and obesity-associated (FTO), and retinol-binding protein. Research has uncovered hundreds of genetic loci associated with obesity, revealing its heterogeneous nature across different populations. Variants in genes like FTO and melanortin-4 receptor (MC4R) have been consistently linked to BMI and fat distribution, highlighting the genetic underpinnings of obesity [269,270]. However, the interaction between genetic susceptibility and environmental factors like diet and physical activity is still not fully understood, leading to significant variability in obesity-related outcomes.

Importantly, many of the genetic loci implicated in obesity also overlap with pathways involved in glucose metabolism, suggesting a shared genetic foundation between obesity and metabolic disorders like T2DM. For example, fto gene variants not only influence BMI but also affect insulin sensitivity and glucose regulation [269]. Similarly, MC4R polymorphisms, which are key regulators of appetite and energy expenditure, have been linked to impaired glycemic control alongside obesity [271]. These findings underscore the interrelated nature of genetic pathways governing both conditions, which can manifest differently depending on an individual’s genetic profile and environmental influences.

### 5.1. Gene-Environment Interactions in Obesity

The heterogeneity of obesity is further influenced by gene-environment interactions. For instance, the *pparγ* gene, which plays a critical role in lipid metabolism and adipogenesis, exhibits variable effects on obesity risk depending on factors such as dietary fat intake [272]. Furthermore, epigenetic modifications, including DNA methylation and histone acetylation, regulate gene expression in response to environmental influences, contributing to obesity [273]. These epigenetic mechanisms also play a role in metabolic outcomes, highlighting the dynamic interplay between genetic predisposition and environmental factors in shaping the health risks associated with obesity.

These findings suggest that obesity’s heterogeneity is not determined by genetics alone, but is also influenced by external factors, including diet and lifestyle. For example, transient hyperglycemia has been shown to induce p65 gene, a regulator of inflammation [274], expression in endothelial cells, which can persist for several days [275]. In addition, obesity risk alleles, such as those in the *fto* gene [276] and *lepr* gene [277], are influenced by diet composition and physical activity levels [276].

### 5.2. Implications for Personalized Medicine and Obesity Management

Understanding the genetic heterogeneity of obesity has profound implications for both clinical practice and public health. Environmental factors can interact with genetically determined glucose homeostasis in genetically susceptible individuals, contributing to the development of obesity and diabetes. Evidence suggests that hyperglycemia can activate genes that perpetuate endothelial dysfunction, a key feature of metabolic disorders [275]. By leveraging insights from genomic studies, clinicians can develop more targeted strategies for managing obesity and its associated complications, such as T2DM.

Genetic profiling could help identify individuals at higher risk for obesity-related metabolic disorders, allowing for earlier and more personalized interventions. Moreover, integrating genomic and epigenetic data into obesity research could offer new therapeutic targets and inform precision medicine approaches. This personalized approach could provide more effective treatments tailored to an individual’s genetic makeup and environmental exposure, ultimately improving health outcomes.

By addressing the genetic diversity of obesity, we can better understand the complex factors that contribute to its development and its association with metabolic diseases, such as T2DM. This understanding is critical for reducing the global burden of obesity-related diseases and improving overall health.

### 5.3. Treatment Options for MODY and Prediabetic Patients

Despite the availability of a variety of pharmacotherapies for glycemic control in T2DM, including biguanides, thiazolidinediones, and sulfonylureas, their major limitation is that they often tend to lose effectiveness after a few years. This suggests that T2DM management remains difficult and challenging, in part due to the genetic variability among patients. These genetic variants are key factors that influence disease susceptibility, response to therapy, and clinical outcomes. Other significant barriers to effective diabetes management include the side effects of current treatments, such as weight gain, hypoglycemia, fluid retention, and gastrointestinal discomfort.

The rising incidence of diabetes, coupled with the limitations of existing medications and their adverse effects, has driven the FDA to approve new antidiabetic drugs almost every year in recent years. This reflects a growing need for more effective, sustainable, and safer treatment options for MODY, adults with pre-diabetes and obesity, or adults who are overweight (Table 2).

While T2DM is a multifactorial disease caused by mutations in various genes, MODY is primarily caused by mutations in genes essential for beta-cell function, leading to defects in insulin secretion. GCK-MODY typically does not require drug therapy, whereas other forms of MODY, such as HNF1A, HNF4A, and KCNJ11, generally respond well to sulfonylurea therapy [278,279]. Sulfonylureas are preferred because they bypass the defective glucose-mediated insulin secretion mechanism [280].

Recent research has explored the efficacy of several medications, including SGLT2 inhibitors, GLP-1 receptor agonists, and glucokinase activators, as alternative or adjunctive therapies for various MODY subtypes, with promising results [281,282,283,284]. Although these medications are not yet approved for MODY treatment, they have shown significant therapeutic potential. Zhao et al. (2024) reported a novel therapeutic approach for GCK-MODY using dorzagliatin, a glucokinase activator [283]. In their case study, dorzagliatin successfully and safely reduced HbA1c levels, highlighting its potential as a targeted therapy for patients with this genetic variant.

Typically, low-dose sulfonylurea medications are effective in treating both HNF1A- and HNF4A-MODY patients, although other oral hypoglycemic agents have also been shown to improve blood glucose control [281]. Patients with heterozygous HNF1A or HNF4A mutations are generally sensitive to sulfonylureas [220]. This suggests that these patients may have an impaired sulfonylurea elimination phase, leading to increased sulfonylurea bioavailability. However, sulfonylurea treatment should be avoided in pregnant carriers of HNF1A and HNF4A mutations.

Sulfonylureas are typically used as the first-line therapy for patients with HNF1A or HNF4A deficiencies. Dipeptidyl peptidase 4 inhibitors or GLP-1 receptor agonists (GLP1-RA) are commonly used as adjunctive therapies for HNF-MODY [220,285]. Moreover, a recently published review highlights the use of GLP1-RA and SGLT2 inhibitors (SGLT2i) as foundational therapies for T2DM and chronic kidney disease (CKD) [286]. For T2DM patients with albuminuria, the AACE guidelines suggest using an angiotensin receptor blocker (ARB) or an ACE inhibitor to reduce the risk of renal disease. Furthermore, they recommend non-steroidal mineralocorticoid receptor antagonists (finerenone) in T2DM kidney disease patients with an eGFR ≥ 25 mL/min/1.73 m^2^, normal serum potassium, and albuminuria (ACR ≥30 mg/g), despite maximum tolerated doses of ARBs or ACE inhibitors, to reduce proinflammatory and profibrotic mediators [287,288]. Finerenone not only reduces albuminuria but has also been found to improve endothelial function and decrease arterial stiffness, thus, lowering the risk of chronic kidney disease in T2DM patients [289].

Almutair and Almulhem (2024) investigated the use of semaglutide, a GLP-1 receptor agonist, in patients with HNF1B-MODY [284]. While semaglutide improved glycemic variability, increased time in range, and decreased HbA1c levels, its adverse effects, such as nausea and appetite suppression, led to early discontinuation. Similarly, Suzuki et al. (2023) examined the role of adjunctive SGLT2i in a patient with HNF4A-MODY [282]. Their study demonstrated that empagliflozin significantly improved glycemic control and lowered HbA1c levels when used alongside sulfonylureas. Unlike GCK-MODY, patients with HNF4A gene variants may develop severe hyperglycemia over time [290], and, thus, may require insulin therapy. If increased insulin sensitivity is observed, diazoxide may be used as an antidote to temporarily suppress insulin release [291].

In conclusion, newer blood glucose-lowering therapies, such as SGLT2i, DPP-4 inhibitors (DPP4i), and GLP-1 receptor agonists (GLP-1RA) have a lower risk of hypoglycemia, and can help with weight loss in individuals who are overweight. Beyond these individual case studies, a recently published review not only focuses on drugs and existing treatment options for MODY, but also emphasizes the importance of precision medicine [281]. Their findings reinforce the necessity of a molecular diagnosis to tailor treatment strategies effectively. Collectively, these studies underscore the potential of emerging therapies in MODY management. While further clinical trials are needed to validate these findings, individualized pharmacologic approaches may revolutionize treatment paradigms for this genetically heterogeneous disorder.

## 6. Future Perspectives

Including data from ethnically diverse populations is essential to ensure that findings on genetic polymorphisms and T2DM treatments are broadly applicable. Studies focused on populations with unique genetic profiles help identify precision diagnostics, develop tailored therapeutic approaches [292], and improve global care and outcomes. Moreover, diverse datasets reduce bias, enabling the development of robust risk prediction models. To achieve this, global collaboration in genomics research and equitable access to clinical trials is crucial. Initiatives like the All of Us Research Program emphasize diverse participation, providing valuable insights into genetic-environmental interactions and advancing personalized medicine frameworks [293,294].

Socioeconomic factors significantly impact access to genetic testing and novel diabetes therapies [295]. Low-income individuals, racial minorities, and those without adequate insurance coverage often face barriers such as high costs, limited availability of advanced healthcare services, and geographic disparities in provider access [296]. For example, newer, high-cost diabetes medications, such as GLP-1 receptor agonists, are more frequently prescribed to individuals with private insurance, while those on Medicaid or without insurance are more likely to rely on older, less effective treatments [296]. Addressing these disparities requires systemic changes, including expanding Medicaid coverage, increasing funding for community health programs, and offering subsidies for genetic testing and advanced therapies [297]. Policies that promote value-based care models can incentivize equitable prescribing practices. Additionally, public awareness campaigns and culturally tailored interventions can help underserved populations [298] better understand and access emerging healthcare technologies, ensuring more equitable treatment outcomes.

Future studies could integrate genetic and environmental data with advanced machine-learning techniques to develop comprehensive risk models for T2DM. Adopting multimodal approaches, as highlighted in several studies, by combining diverse data types such as SNPs, gene expression profiles, and environmental factors (e.g., diet and physical activity) can enhance the predictive accuracy of these models [299,300]. This multimodal strategy offers a holistic understanding of gene–environment interactions, which are pivotal in T2DM development. Deep learning models, known for their ability to capture non-linear and complex relationships, are particularly well-suited for integrating high-dimensional genetic data with real-time lifestyle data from wearable devices or health apps. Furthermore, leveraging large-scale biobanks and global cohorts, as emphasized by Mohsen et al. (2023), can provide diverse datasets that reflect genetic and environmental variability across populations [299]. By combining genetic markers with modifiable lifestyle factors (Figure 1), researchers can create dynamic risk prediction models that enable personalized interventions, ultimately improving prevention and management strategies for T2DM.

The scoping review by Mohsen et al. (2023) underscores the advancements in bioinformatics and machine learning (ML) that can significantly enhance the analysis of complex genetic data to predict therapeutic responses in T2DM [299]. EHR has proven to be a promising tool for studying complex, multifactorial diseases like T2DM [301], by providing a more comprehensive understanding of disease mechanisms, as well as the clinical management of T2DM and its complications. Although deep learning models are still underrepresented compared to traditional ML approaches, they have shown great potential in handling high-dimensional genomic datasets. Incorporating interpretability techniques further enhances our understanding of the genetic determinants driving therapeutic responses. Additionally, leveraging external validation and open-source tools can improve the strength and reproducibility of ML models. Advancements in integrating lipidomic and metabolomic data, as well as biomarkers that reliably predict individual risk, remain underutilized but offer valuable insights into metabolic pathways linked to drug efficacy [302,303].

Complications of T2DM (Figure 3) arise from multiple factors (Figure 1). As discussed earlier, chronic stressors contribute to accelerated tissue damage and disruption. These stresses have varied effects depending on tissue, cellular, and molecular milieu. For instance, the tcf7l2rs7903146 gene variant [304] and tcf7l2rs290487 gene variant [173] are linked to a decreased response to sulfonylurea medications and higher HbA1C levels in cirrhotic diabetic patients, respectively, producing different sources of tissue and cellular stresses. By combining innovations, large-scale genomic datasets representing the full diversity of the U.S. across ancestries, ethnicities, genders, and biobank-powered studies, we can advance precision medicine for T2DM.

To overcome adherence challenges, healthcare providers should prioritize patient-centric strategies such as simplified regimens (e.g., once-weekly injectable GLP-1 receptor agonists) [305]. Financial support programs, including copay assistance or value-based pricing, can alleviate cost burdens [306]. Behavioral interventions, like motivational interviewing and shared decision-making, can help align treatments with patient preferences and improve adherence [307]. Furthermore, telehealth services offer remote follow-ups, continuous support, and glucose control [308], while mobile apps allow patients to track medication adherence and glucose levels [309]. Tailored education addressing cultural and socioeconomic barriers can further reduce nonadherence in diverse populations [310].

Barriers to effective glycemic control exist at the patient, healthcare system, health insurance status, and prescriber levels [311,312]. Healthcare systems must adopt a multifaceted approach to address gaps in glycemic control. Implementing continuous glucose monitoring and digital health tools can provide real-time feedback to patients and clinicians, leading to better glycemic control, particularly in cases of uncontrolled diabetes [313]. Lastly, patient education and support programs are vital to improving medication adherence and promoting lifestyle changes [310]. Expanding access to affordable medications and reducing barriers to advanced therapies through insurance coverage or subsidy programs can further bridge this gap, especially for underserved populations. Collaboration across stakeholders—clinicians, policymakers, and payers—is essential to optimizing diabetes care delivery.

Genetic studies have significantly advanced our understanding of T2DM by identifying numerous novel susceptibility loci, which have helped improve T2DM risk prediction. This genetic information also provides valuable insights into how to assess individual variability in response to glucose-lowering agents. Many pharmacogenetic studies have investigated the associations between genetic variants and the response to glucose-lowering drugs. While hundreds of SNPs are found in non-coding regions of genes that do not translate into proteins, certain polymorphisms can affect the gene in ways that reduce protein activity, leading to variability in drug efficacy. For example, genetic changes can produce dysfunctional proteins or prevent their production altogether, which can influence the effectiveness of drugs like metformin or sulfonylureas.

One promising area is the development of drugs that could target ATP-sensitive potassium (KATP) channels in pancreatic beta cells in T2DM patients carrying the TCF7L2 rs7903146 gene variant. This highlights how knowledge of specific SNPs can potentially improve treatment outcomes and reduce T2DM-related complications.

However, several barriers remain to fully utilize SNPs in clinical practice. These include ethical concerns, patient willingness to undergo genetic testing, the lack of standardized sequencing data for clinical use, and the limited motivation among pharmaceutical companies to develop drugs targeting specific genetic changes, particularly for a relatively small subset of T2DM patients globally. While these barriers are gradually being addressed, they still represent significant challenges in translating genetic research into effective, personalized treatments.

## 7. Conclusions

The genetic basis of T2DM is multifaceted, involving both monogenic and polygenic forms. When uncontrolled, T2DM progresses into a multisystemic condition, leading to microvascular and macrovascular complications, clotting abnormalities, renal damage, inflammation, hypertension, retinopathy, and peripheral neuropathy. These complications contribute to significant morbidity and disability for patients with T2DM worldwide.

Recent advancements in T2DM treatment, including new DPP-4 inhibitors, oral small-molecule GLP1-RAs, and newer options for SGLT2i, have shown promise in improving diabetes management. These treatments are particularly beneficial for addressing obesity, which is a major risk factor for T2DM.

GWAS have identified hundreds of loci associated with T2DM by examining common genetic variations. Notably, the GLIS3 gene is strongly linked to T2DM and may serve as a potential marker for endothelial function. GWAS findings have revealed novel genes and therapeutic targets for managing T2DM. This review highlights both the monogenic and polygenic contributions to uncontrolled T2DM, with a special focus on genes such as PPARγ, Lep, UCP1, and OPG, which influence insulin delivery and glucose uptake in tissues. These genes interact across various pathways to impact blood glucose regulation.

Moreover, the review explores the roles of genes that contribute to beta-cell dysfunction, impaired insulin secretion, and vascular complications associated with diabetes, offering insights into their genetic and epigenetic mechanisms. Ongoing research into these genes will expand our understanding of T2DM and aid in the development of more effective strategies to combat this global epidemic.

## Figures and Tables

**Table 1:** Summary of multiple GWAS across different populations consistently shows associations between certain codon variants in specific genes and MODY or T2DM.

Genes	Role of Gene Product	Rare Variants	Phenotypic Abnormalities	References
	The most potential pathogenic genes in MODY			
*Gck*	Glucokinase catalyzes the rate-limiting step of the glycolysis pathway, functions as the glucose sensor in pancreatic beta cells	c.1015del, p.(glu339Argfs*14) p.G72R, C220Y, p.L58P, p.F123S	Mild hyperglycemia, overweight Glycemic	[32,33,34]
*Hnf1a*	Hepatic nuclear factor 1-alpha is critical for the growth and development of beta cells in the pancreas, controls genes involved in liver development, regulates cell growth and survival, and acts as a tumor suppressor.	R200C T196A P291fsinsC H126D	Decreased insulin secretion Increased hepatogenic secretion of atherogenic lipoproteins Liver steatosis Decreased glucose transporter GLUT2 expression, reduced ATP production	[35,36,37,38]
*Hnf4a*	Hepatic nuclear factor 4-alpha controls genes that are important for the development and function of beta cells in the pancreas.	rs4735692 V393I c.110T>c, c.1097C>G p.R114W	Associated with obesity Reduced transcriptional activity and insulin secretion Increases risk of diabetes	[39,40,41,42]
*Kcnj11*	Encodes pore-forming inwardly-rectifying potassium channel subunits (Kir6.2)	rs5215, rs5218, and rs5219	MODY, T2DM, elevated FBS, high BMI	[43,44,45,46]
The most significant genes that might play an essential role in late-onset T2DM				
*tcf7l2*	A key regulator of insulin secretion in pancreatic beta-cells	rs7903146	T2DM, less obesity, lower insulin secretion and higher insulin action at diabetes onset	[47,48,49]
*glis3*	A transcription factor: Regulator of islet development, insulin gene transcription, and obesity-induced compensatory β-cell proliferation	P/LP GLIS3, rs10758593 rs 7034200	Neonatal diabetes mellitus, T2DM congenital hypothyroidism and polycystic kidney	[50,51,52]
*pparγ*	A transcription factor: master regulator of adipogenesis, energy balance, lipid biosynthesis, and insulin sensitivity; cellular target of TZDs	rs4684847 rs1801282	T2DM, impaired insulin sensitivity, partial lipodystrophy	[53,54,55]
*lep*	A regulator of appetite and energy balance	rs147287548 G2548A	T2DM, severe obesity, hyperglycemia	[42,56]
*ucp1*	A regulator of energy balance and mitochondrial-induced oxidative stress	rs45539933	T2DM, increased body fat accumulation, risk of insulin resistance	[57,58,59]
*opg*	A decoy receptor for receptor-activator for NF-κB ligand (RANKL)	rs2073618	T2DM, inflammation, hypertension	[60]

**Table 2:** Medications for maturity-onset diabetes of the young and prediabetes between 2020 and 2025.

Conditions	Medicines	MAO	Outcomes	Side Effects	Comments
Adjunctive medications for MODY					
GCK-MODY	Dorzagliatin	A glucokinase activator	Improves glycemic control	Potential gastrointestinal issues, hypoglycemia, and headaches	
Heterozygous loss of function in *Hnf1a*	Low-dose sulfonylurea	Stimulate insulin secretion from the pancreas	Improves glycemic control	Hypoglycemia	
Homomozygous *Hnf1a* mutations	There are currently no approved treatments.				
HNF1B-MODY	SGLT2 inhibitors Semaglutide Insulin	Increase glycosuria A GLP-1 receptor agonist A hormone	Lowers blood glucose levels Helps manage blood sugar levels Regulates blood sugar levels	Increased urination frequency and urinary tract infection Diarrhea hypoglycemia	
HNF4A-MODY	Empagliflozin	SGLT2 inhibitor	Improves glycemic control		
The FDA approved new treatments for adults with pre-diabetes and obesity or overweight					
T2DM	Brenzavvy (bexagliflozin)	A dual agonist for the glucagon-like peptide-1 (GLP-1) and glucose-dependent insulinotropic polypeptide (GIP) receptors	Improves hyperglycemia and promotes weight loss	It may cause ketoacidosis, a serious, potentially life-threatening complication that occurs when the body produces high levels of acids in the blood	It works similarly in Asian, Black or African American, and White adults
T2DM	Mounjaro (tirzepatide)	Selectively binds to and activates both the GIP and GLP-1 receptors to target GIP and GLP-1, the native incretin hormones	Improves blood sugar control, promotes weight loss	It may cause serious side effects including inflammation of the pancreas (pancreatitis), low blood sugar, allergic reactions, kidney problems (kidney failure), severe stomach problems, and complications of diabetes-related eye disease (diabetic retinopathy)	It works similarly in Asian, Black or African American, and White adults
CKD associated T2DM	Kerendia (finerenone)	Blocks the mineralocorticoid receptor	It reduced the risk of kidney failure associated with T2DM, having a heart attack or stroke, being hospitalized for heart failure, and dying from cardiovascular disease	The most common side effects included high potassium levels in the blood (hyperkalemia), low blood pressure (hypotension), and low sodium levels in the blood (hyponatremia).	No notable difference in side effects was observed by racial subgroups.

## Data Availability

The original contributions to the study are included in the article, further inquiries can be directed to the corresponding author.

## Abbreviations

The following abbreviations are used in this manuscript:

ADA	American Diabetes Association
BMI	Body mass index
CV	Cardiovascular
CNS	Central nervous system
DPP-4	dipeptidyl peptidase-4
*FTO*	Fat mass and obesity-associated
FDA	Food and Drug Administration
*GLIS3*	Gli-similar 3
GLP-1	Glucagon-like peptide-1
GIP	Glucose-dependent insulinotropic polypeptide
GWAS	Genome-wide association studies
*HNF4α*	hepatocyte nuclear factor 4-alpha
HbA1C	Plasma glycosylated hemoglobin A1C
MC4R	Melanortin-4 receptor
MeSH	Medical subject headings
MODY	Maturity-onset diabetes of the young
NF-κB	Nuclear factor-κB
*OPG*	Osteoprotegerin
PPARγ	Peroxisome proliferator-activated receptor Gamma
SNPs	Single nucleotide polymorphisms
SGLT2	Sodium-glucose co-transporter-2
T2DM	Type 2 diabetes mellitus
TGFβ	Transforming growth factor beta
WHO	World Health Organization
